# Supplementary material for: Gut microbiota aggravates neutrophil extracellular traps-induced pancreatic injury in hypertriglyceridemic pancreatitis
Source: Nat Commun. 2023 Oct 4;14:6179. doi: 10.1038/s41467-023-41950-y (PMC10550972; doi:10.1038/s41467-023-41950-y)
Supplement: Supplementary file 1 — Supplementary information [file 41467_2023_41950_MOESM1_ESM.pdf]

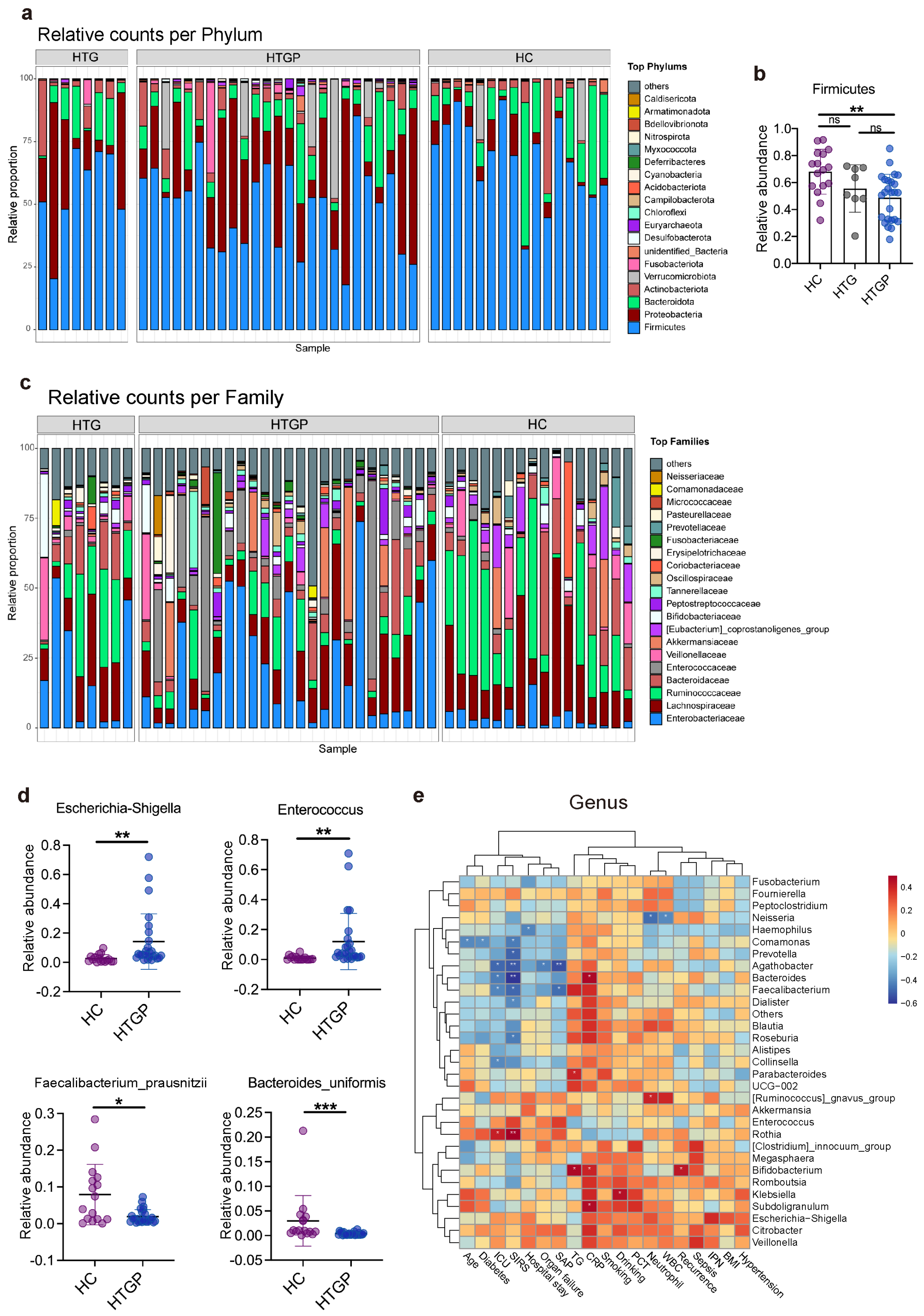

Supplementary Fig.1 Gut microbiota differs between HTGP patients and healthy volunteers.

a. Relative abundance of gut microbiota in the phyum level. Top 20 abundant and prevalent phyla were visualized (healthy volunteers, n=16; high triglycerides matched controls, n=8; HTGP patients,

n=25). **b.** The relative abundance of *Firmicutes* between healthy volunteers and HTGP patients. **c.** The relative abundance of bacteria at family level in each sample. **d.** The relative abundance of *Escherichia Shigella, Enterococcus, Faecalibacterium prausnitzii* and *Bacteroides uniformis* between HC and HTGP patients (healthy volunteers, n=16; HTGP patients, n=25). **e.** Correlation analysis between different genera and clinical outcomes in HTGP patients. Normalized values were analyzed by Student's *t*-test to compare two experimental groups or by ANOVA with the Tukey post hoc test to compare more than two groups in parallel. Data was represented as mean ± SEM. *$p<0.05$, **$p<0.01$, ***$p<0.001$. Source data are provided as a Source data file.

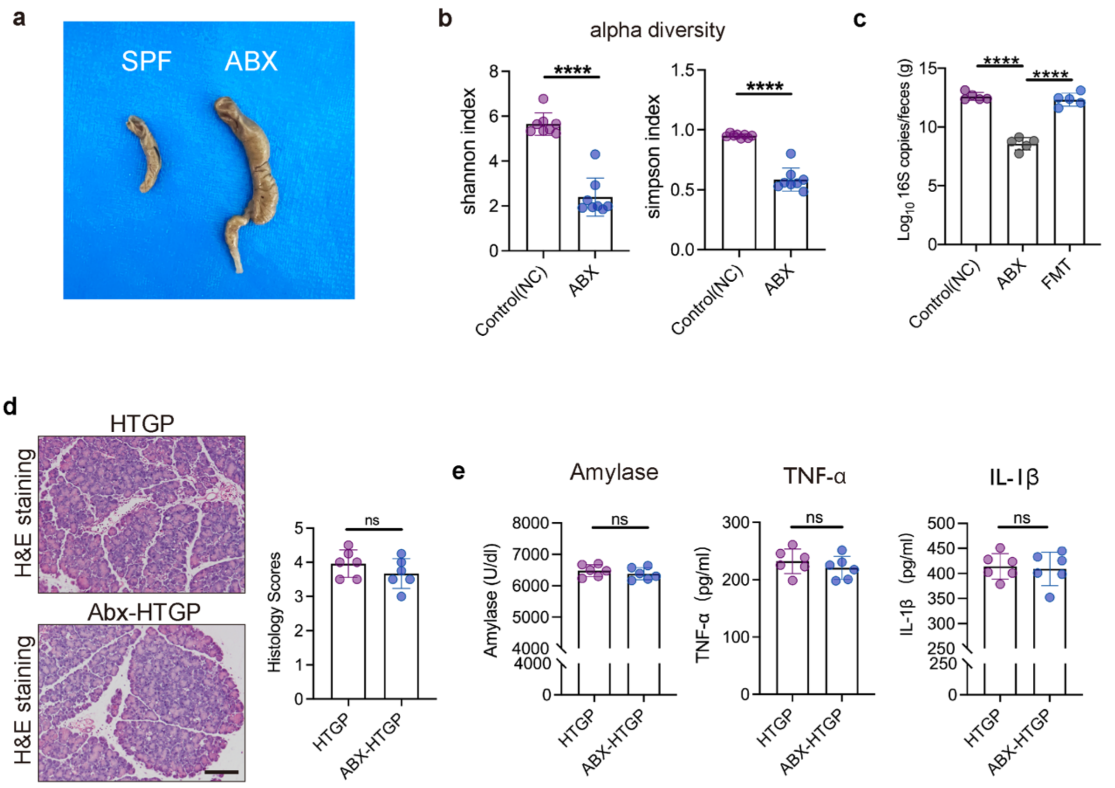

Supplementary Fig.2 Gut microbiota is depleted by antibiotic solution.

a. Cecum dissected from PBS-treated or ABX-treated mice. **b.** Alpha diversity between control and ABX-treated mice (based on shannon and simpson, Control, mice were reared in SPF condition, n=8; ABX, mice treated with a broad-spectrum antibiotic treatment, n=8). **c.** The 16S rDNA copies/feces (g) was detected by qRT-PCR. **d.** Representative images of H&E staining between control and ABX-treated HTGP mice (Scale bar=100 μm, n=6). **e.** Serum amylase, TNF-α and IL-1β levels (n=6). Normalized values were analyzed by Student's *t*-test to compare two experimental groups. Data was represented as mean ± SEM. **p*<0.05, ***p*<0.01, ****p*<0.001. Source data are provided as a Source data file.

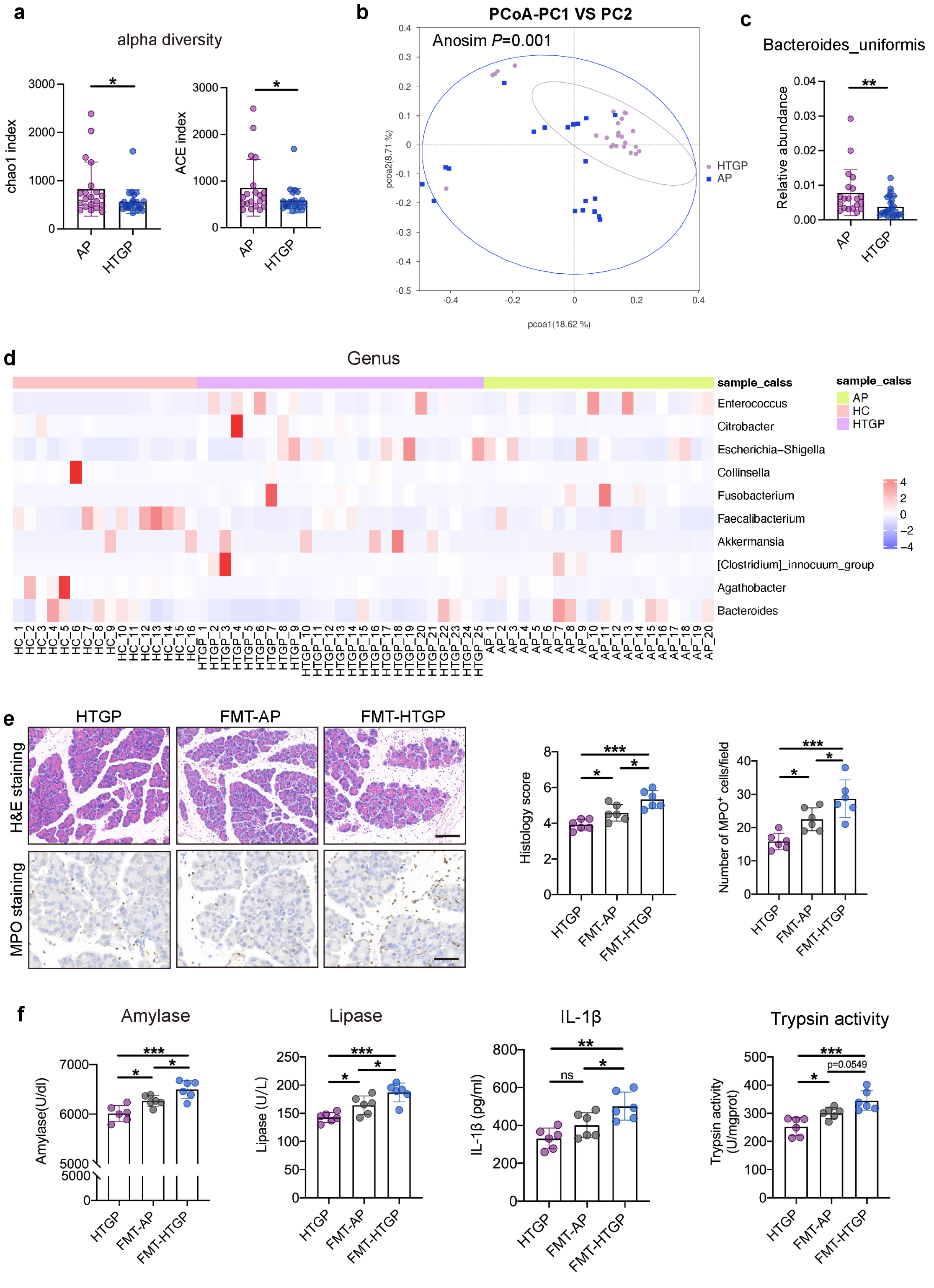

Supplementary Fig.3 HTGP-induced imbalanced gut microbiota exacerbates pancreatic injury.

a. Alpha diversity between AP patients and HTGP patients (based on chao1 and ACE, AP, acute pancreatitis caused by other etiologies, n=20; HTGP, hypertriglyceridemic pancreatitis, n=25). b. Principal coordinate analysis (PCoA) analysis showing beta diversity by unweighted unifrac metric

distance. **c.** The abundance of *Bacteroides uniformis* between AP and HTGP patients (AP, n=20; HTGP, n=25). **d.** The hierarchical clustering heatmap of differentially expressed genus among HTGP patients, AP patients and healthy volunteers (Top 10 abundance of microbes were compared). **e.** Representative images of H&E staining and Mpo positive cells by IHC among HTGP, FMT-AP and FMT-HTGP groups (H&E staining, Scale bar=100 μm, n=6; IHC, Scale bar=50 μm, n=6). **f.** Serum amylase, lipase and IL-1β levels and pancreatic trypsin activity (n=6). For intergroup comparison of unpaired data, Student's *t*-test was used for normal distribution and Mann-Whitney test was used for nonparametric data. Data was represented as mean ± SEM. *$p<0.05$, **$p<0.01$, ***$p<0.001$. Source data are provided as a Source data file.

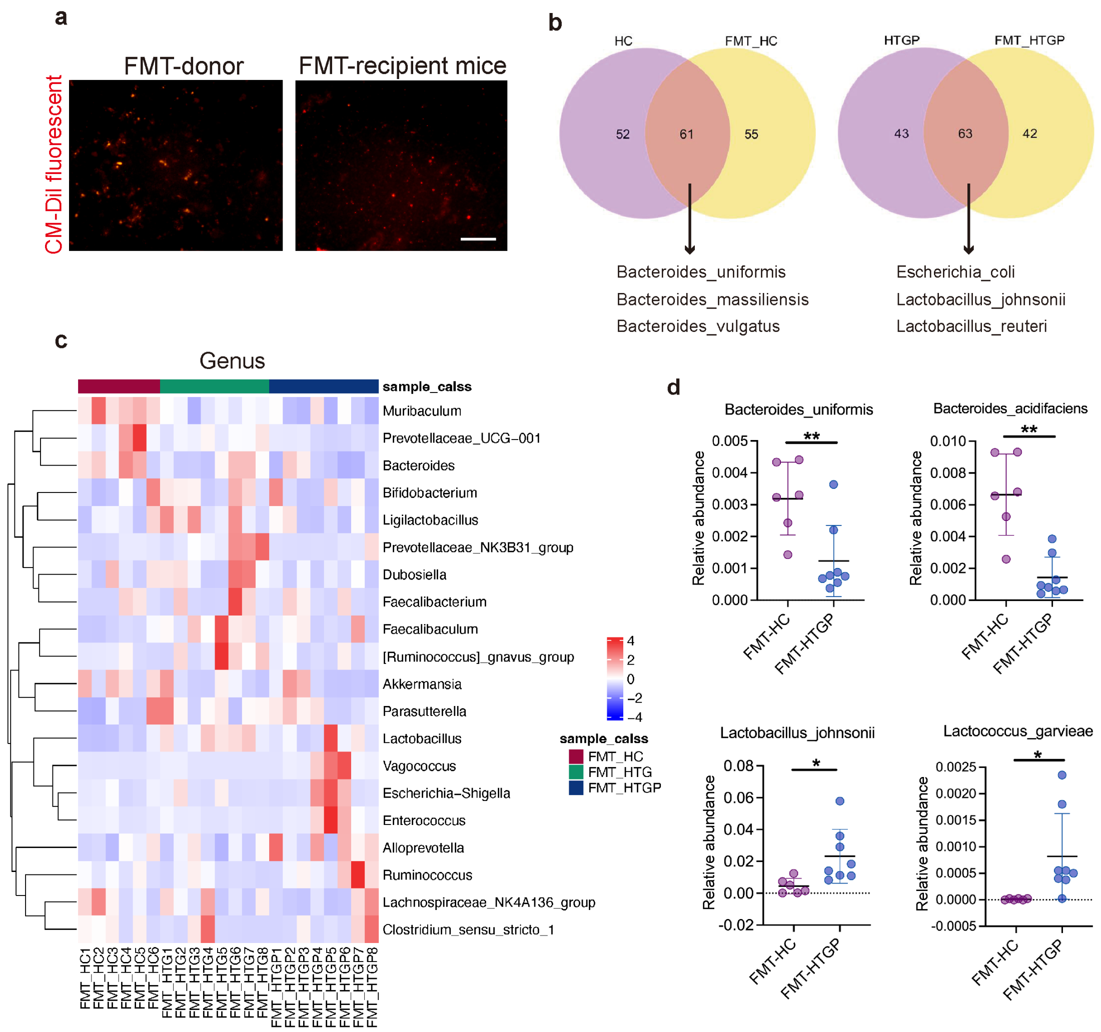

Supplementary Fig.4 FMT reshapes gut microbiota in recipient mice.

a. Staining of live bacteria in fecal samples obtained from donors and FMT-recipient mice. **b.** The bacterial species detected from donors and recipient mice were intersected by Venn diagram. **c.** The hierarchical clustering heatmap showing different genera among FMT-HC, FMT-HTG and FMT-HTGP groups. **d.** Relative abundance of bacterial species between FMT-HC, and FMT-HTGP groups (FMT-HC, n=6; FMT-HTGP, n=8). For intergroup comparison of unpaired data, Student's *t*-test was used for normal distribution and Mann-Whitney test was used for nonparametric data. Data was represented as mean ± SEM. **p*<0.05, ***p*<0.01, ****p*<0.001. Source data are provided as a Source data file.

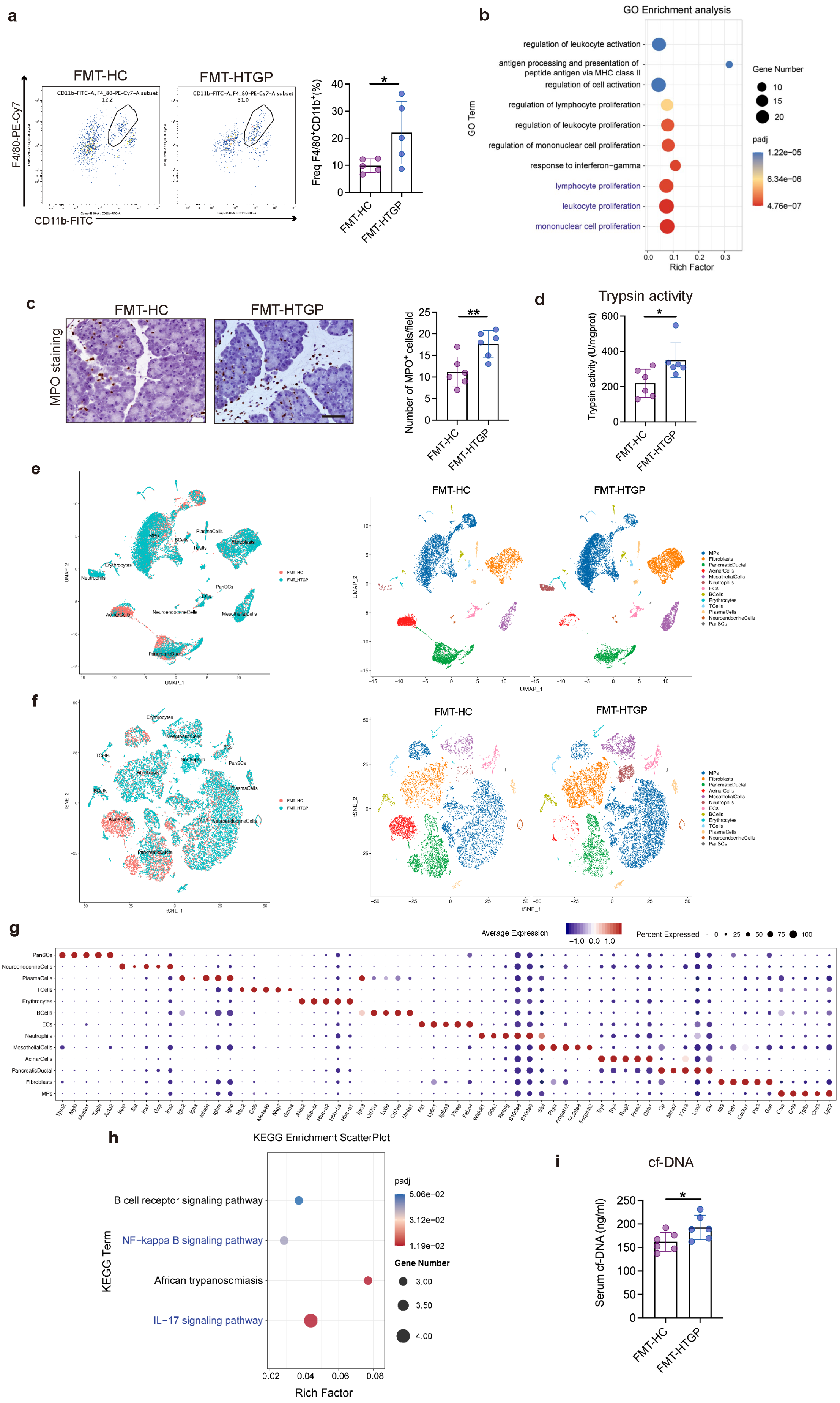
a
FMT-HC
FMT-HTGP
F4/80-PE-Cy7
CD11b-FITC
Freq F4/80+CD11b+(%)
*
FMT-HC
FMT-HTGP
b
GO Enrichment analysis
regulation of leukocyte activation
antigen processing and presentation of peptide antigen via MHC class II
regulation of cell activation
regulation of lymphocyte proliferation
regulation of leukocyte proliferation
regulation of mononuclear cell proliferation
response to interferon−gamma
lymphocyte proliferation
leukocyte proliferation
mononuclear cell proliferation
GO Term
Rich Factor
Gene Number
10
15
20
padj
1.22e−05
6.34e−06
4.76e−07
c
FMT-HC
FMT-HTGP
MPO staining
Number of MPO+ cells/field
**
d
Trypsin activity
Trypsin activity (U/mgprot)
*
e
FMT-HC
FMT-HTGP
UMAP_1
UMAP_2
f
FMT-HC
FMT-HTGP
tSNE_1
tSNE_2
g
Average Expression
Percent Expressed
h
KEGG Enrichment ScatterPlot
B cell receptor signaling pathway
NF−kappa B signaling pathway
African trypanosomiasis
IL−17 signaling pathway
KEGG Term
Rich Factor
padj
5.06e−02
3.12e−02
1.19e−02
Gene Number
3.00
3.50
4.00
i
cf-DNA
Serum cf-DNA (ng/ml)
*
FMT-HC
FMT-HTGP

Supplementary Fig.5. FMT reduces immune activation and ameliorates pancreatic injury in HTGP. **a.** Flow cytometric quantification and statistical analysis of infiltrated macrophages in the pancreas between FMT-HC and FMT-HTGP groups (n=5). **b.** Gene ontology (GO) analysis showing top 10 pathways (biological functions) enriched by up-regulated genes in FMT-HTGP group (FMT-HC, n=3; FMT-HTGP, n=3). **c.** Representative image and quantification of Mpo positive cells between FMT-HC and FMT-HTGP groups (Scale bar=50μm; n=6). **d.** Intrapancreatic trypsin activation between FMT-HC mice and FMT-HTGP mice (n=6). **e.** Overview of the single-cell transcriptional profiling of cells isolated from pancreas using UMAP. **f.** Overview of the single-cell transcriptional profiling of pancreatic cells using tSNE. **g.** Dot plot showing scaled signature genes in each cluster, colored by average gene expressions in each cluster. Dot size represents the percentage of cells in each cluster. **h.** KEGG analysis visualizing the upregulated pathways of neutrophils in FMT-HTGP group (covered gene number ≥ 3, adjusted p values ≤ 0.05). **i.** Quantification of NETs release by measuring serum DNA levels (n=6). Normalized values were analyzed by Student's *t*-test to compare two experimental groups or by ANOVA with the Tukey post hoc test to compare more than two groups in parallel. Data was represented as mean ± SEM. *p<0.05, **p<0.01, ***p<0.001. Source data are provided as a Source data file.

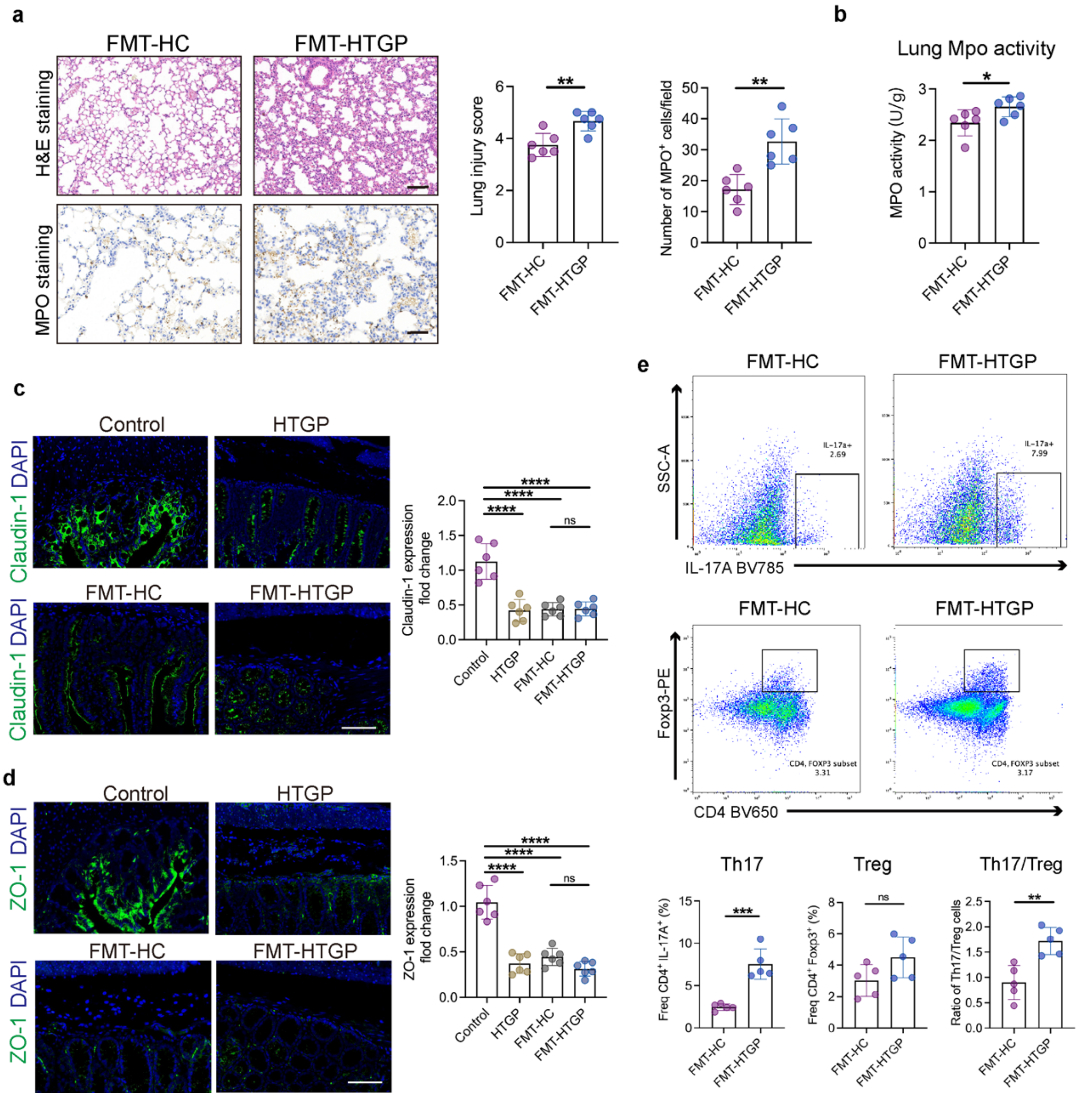

Supplementary Fig.6 FMT affects intestinal barrier and gut immune system.

a. Representative images of H&E staining and Mpo positive cells in lung between FMT-HC and FMT-HTGP groups (H&E staining, Scale bar=100 μm, n=6; IHC, Scale bar=50 μm, n=6). **b.** Lung Mpo content of mice colonized with gut microbiota from HC and HTGP donors (n=6). **c.** Representative images of immunofluorescence with antibodies against Claudin-1 in the colon (n=6). **d.** Representative images of immunofluorescence with antibodies against ZO-1 in the colon (n=6). **e.** Representative staining of IL-17A^{+}CD4 T cells and FOXP3^{+} CD4 T cells subsets and statistical analysis of the percentages in the lamina propria of colon (n=5). Normalized values were analyzed by Student's *t*-test to compare two experimental groups or by ANOVA with the Tukey post hoc test to compare more than two groups in parallel. Data was represented as mean ± SEM. **p*<0.05, ***p*<0.01, ****p*<0.001. Source data are provided as a Source data file.

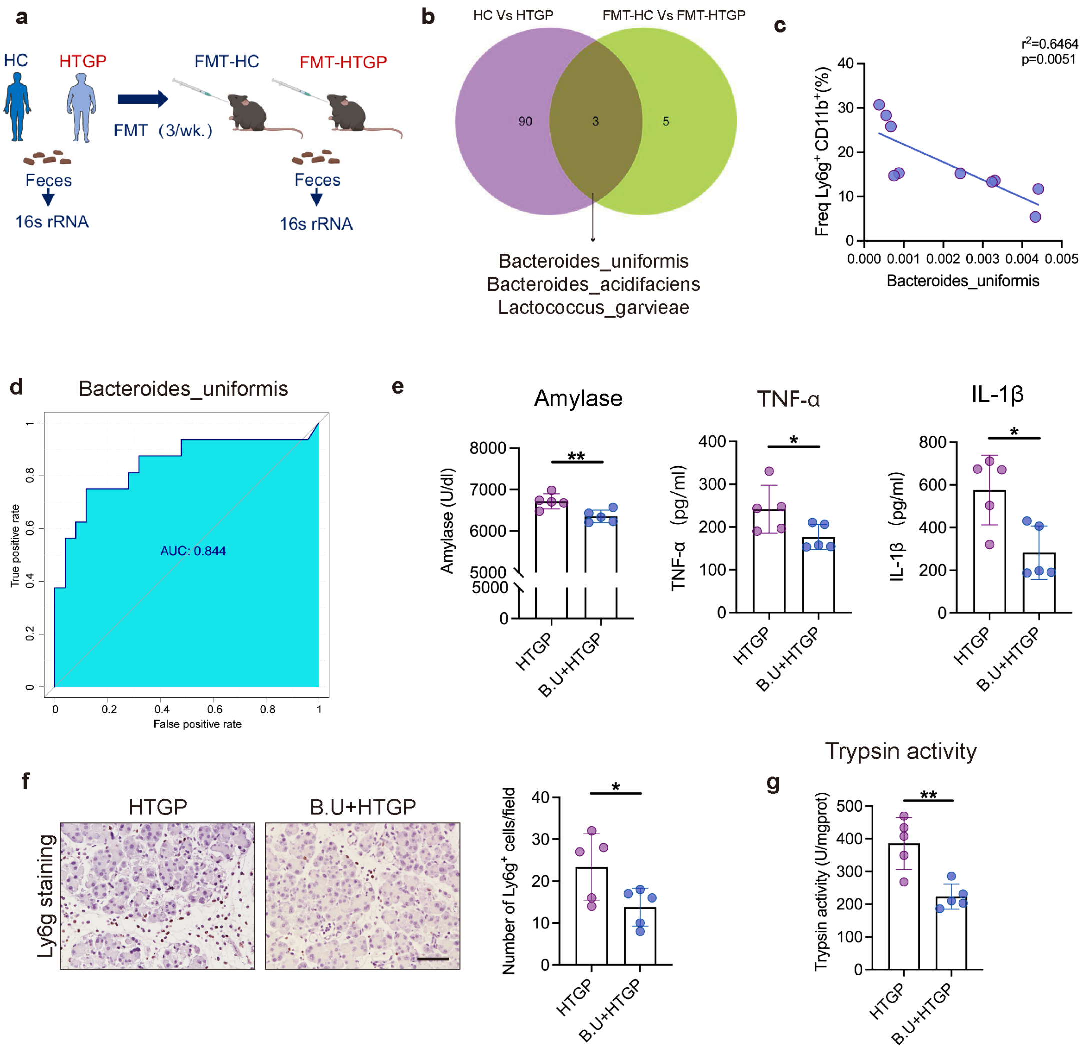

Supplementary Fig.7 *Bacteroides uniformis* suppresses neutrophil recruitment and alleviates HTGP.

a. Schematic representation for FMT study. **b.** Venn diagram showing differential bacterial species intersected between donors and recipient mice. **c.** The correlation analysis between *Bacteroides uniformis* abundance and neutrophils infiltration. **d.** Receiver operating curve (ROC) for *Bacteroides uniformis* in human cohort (HTGP patients, n=25; healthy volunteers, n=16). The area under the receiver operating curve (AUC) is 0.844. **e.** Serum amylase, IL-1β and TNF-α levels between HTGP and B.U-treated HTGP mice (P407-induced HTGP model, n=5). **f.** Representative image and quantification of Ly6g staining between control and B.U-treated HTGP mice (P407-induced HTGP model, scale bar=50 μm, n=5). **g.** Intrapancreatic trypsin activation between HTGP and B.U-treated HTGP mice (n=5). Normalized values were analyzed by Student's *t*-test to compare two experimental groups. Data was represented as mean ± SEM. *p<0.05, **p<0.01, ***p<0.001.

Source data are provided as a Source data file.

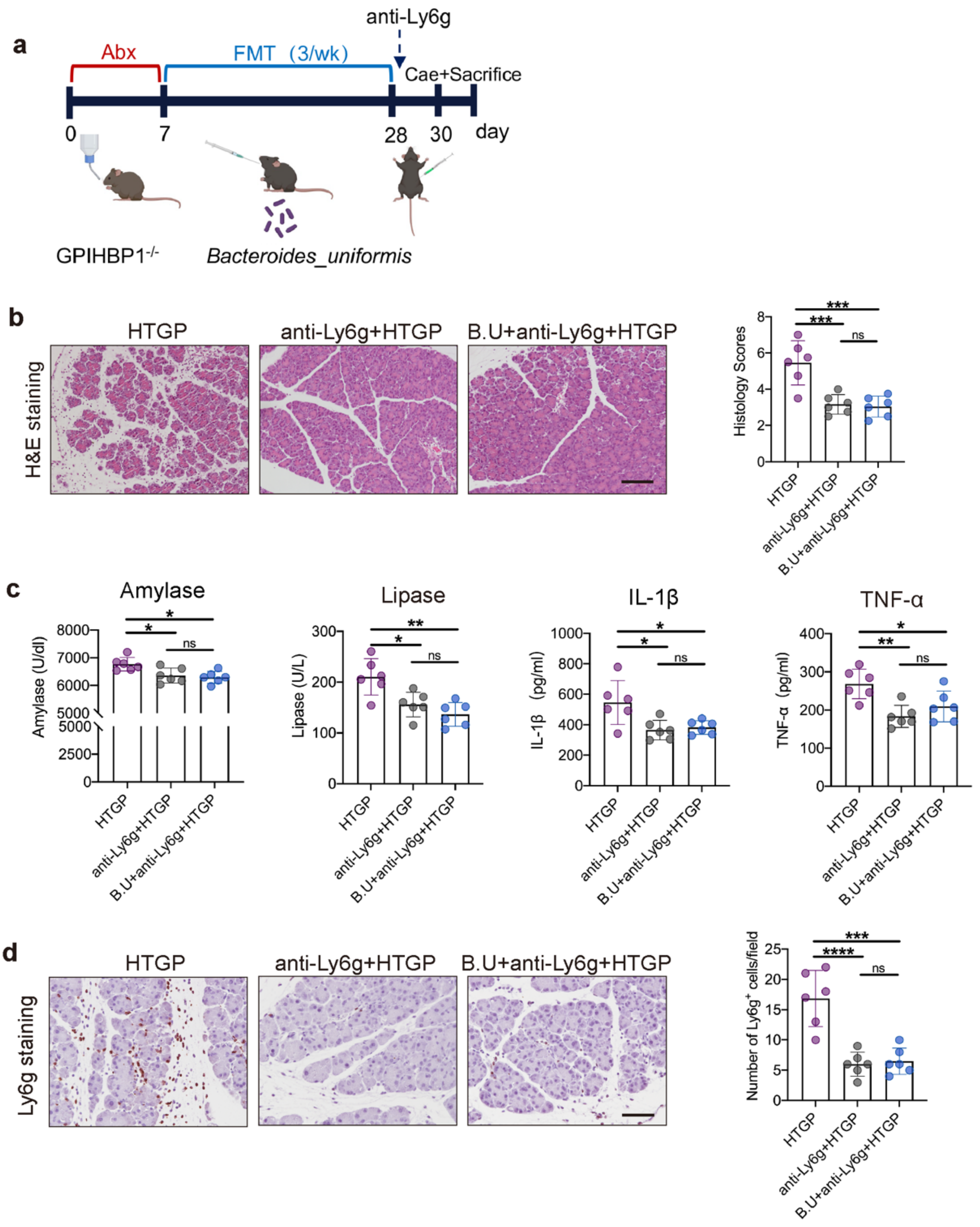

Supplementary Fig.8 *Bacteroides uniformis* alleviates HTGP through direct neutrophil inhibition. a. Schematic representation of *Bacteroides uniformis* administration study. GPIHBP1$^{-/-}$ mice were randomly divided into the following three groups (control, Ly6g neutralization, and B.U combined with Ly6g neutralization), and HTGP model was built by Cae-injection (n=6 per group). **b.** Representative images of H&E staining and quantification of histology score (Scale bar=100 μm) **c.** Serum levels of amylase, lipase, TNF-α and IL-1β. **d.** Representative image and quantification of Ly6g positive cells in control, anti-Ly6g and B.U+anti-Ly6g groups (Scale bar=50 μm, n=6). One-way ANOVA with the Tukey post hoc test to compare more than two groups in parallel. Data was represented as mean ± SEM. **p*<0.05, ***p*<0.01, ****p*<0.001. Source data are provided as a Source data file.

a Metabolism pathway

Pathway	Total	Hits	Raw p	-ln(p)	Impact	Hits Cpd
Taurine and hypotaurine metabolism	8	1	0.038938	3.2458	0.42857	Taurine cpd:C00245
Primary bile acid biosynthesis	46	2	0.019519	3.9364	0.02976	Taurine cpd:C00245; Cholic acid cpd:C00695
Histidine metabolism	15	1	0.071936	2.632	0.24194	L-Histidine cpd:C00135
Glycine, serine and threonine metabolism	31	1	0.14373	1.9398	0.03141	Guanidoacetic acid cpd:C00581
Arginine and proline metabolism	44	1	0.19851	1.6169	0.02333	Guanidoacetic acid cpd:C00581
Nitrogen metabolism	9	1	0.043713	3.1301	0	L-Histidine cpd:C00135
Aminoacyl-tRNA biosynthesis	69	1	0.29545	1.2192	0	L-Histidine cpd:C00135

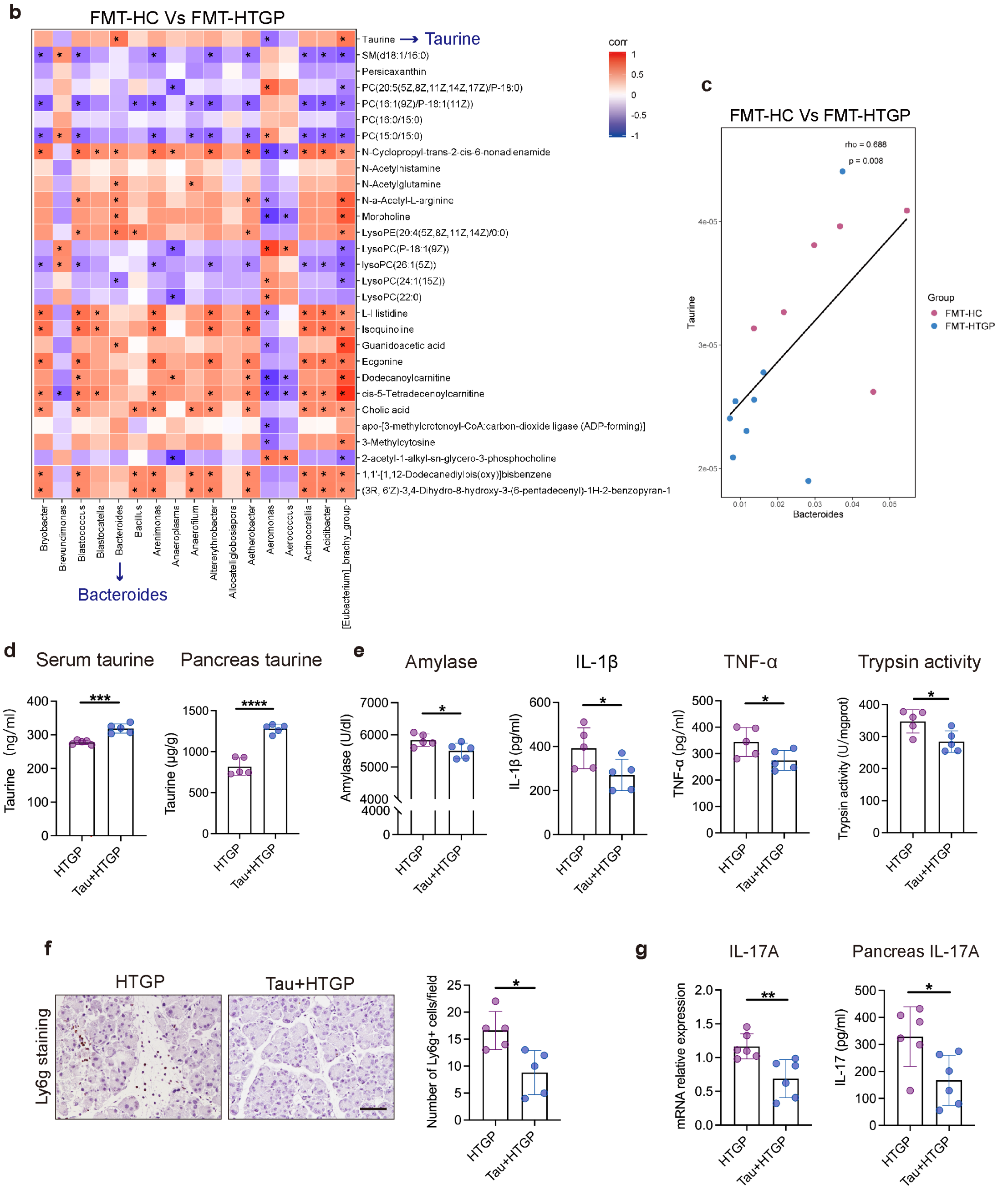

Supplementary Fig.9 Associations between altered gut bacteria and different circulating metabolites.

a. Taurine metabolism was revealed as top pathway in metabolic pathway analysis between FMT-HC and FMT-HTGP mice. **b.** Correlation analysis between altered gut microbiota and differentially changed metabolites between FMT-HC and FMT-HTGP groups. **c.** Correlation analysis between fecal *Bacteroides*

abundance and serum taurine level in FMT-HC and FMT-HTGP groups. **d.** Comparison of serum and pancreas taurine levels between HTGP and taurine-treated HTGP groups (Gpihbp1$^{-/-}$ mice-induced HTGP, n=5). **e.** Serum amylase, IL-1β and TNF-α levels were tested by ELISA assay and intrapancreatic trypsin activation was compared between HTGP and taurine-treated HTGP groups (n=5). **f.** Representative image and quantification of Ly6g positive cells between HTGP and taurine-treated groups (Scale bar=50 μm, n=5). **g**. Quantitative RT-PCR and ELISA tested IL-17A mRNA expression and protein level in the pancreas (n=6). For intergroup comparison of unpaired data, Student's *t*-test was used for normal distribution. Data was represented as mean ± SEM. *$p<0.05$, **$p<0.01$, ***$p<0.001$. Source data are provided as a Source data file.

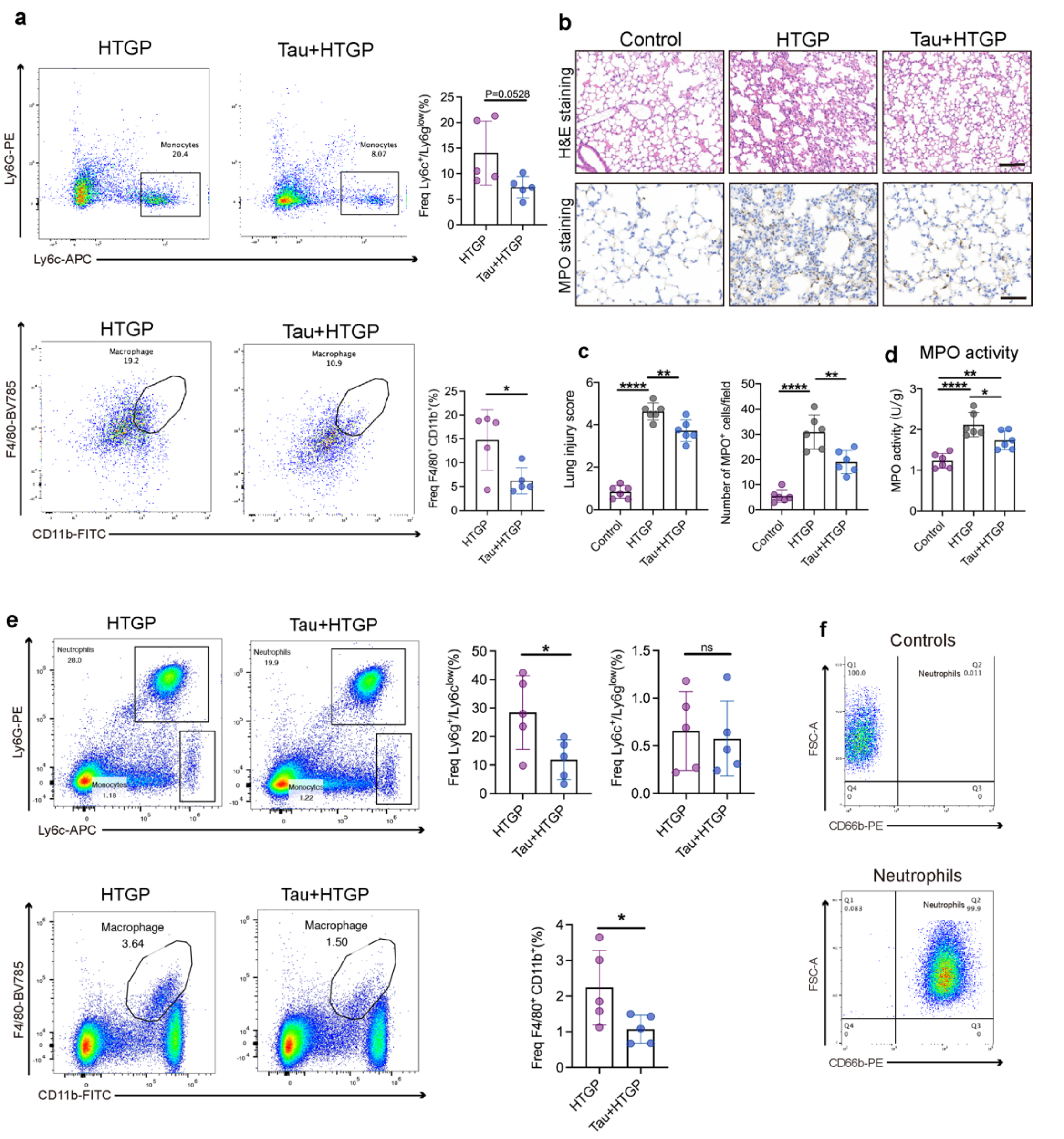

Supplementary Fig.10 Taurine suppresses systemic inflammation and alleviates HTGP.

a. Flow cytometric quantification and statistical analysis of infiltrated macrophages and monocytes in the pancreas between Control and taurine-treated groups (n=5). **b.** Representative images of H&E staining and Mpo positive cells in the lung by IHC (H&E staining, Scale bar=100 μm, n=6; IHC, Scale bar=50 μm, n=6). **c.** Quantification of histology score and Mpo positive cells by IHC among Control, HTGP and taurine-treated groups (n=6). **d.** Lung Mpo activity (n=6). **e.** Comparison of systemic immune modulation between HTGP and taurine-treated groups (n=5). **f.** The purity of neutrophils was detected by flow cytometry. Normalized values were analyzed by Student's *t*-test to compare two experimental groups or by ANOVA with the Tukey post hoc test to compare more than two groups in parallel. Data was represented as mean ± SEM. **p*<0.05, ***p*<0.01, ****p*<0.001. Source data are provided.

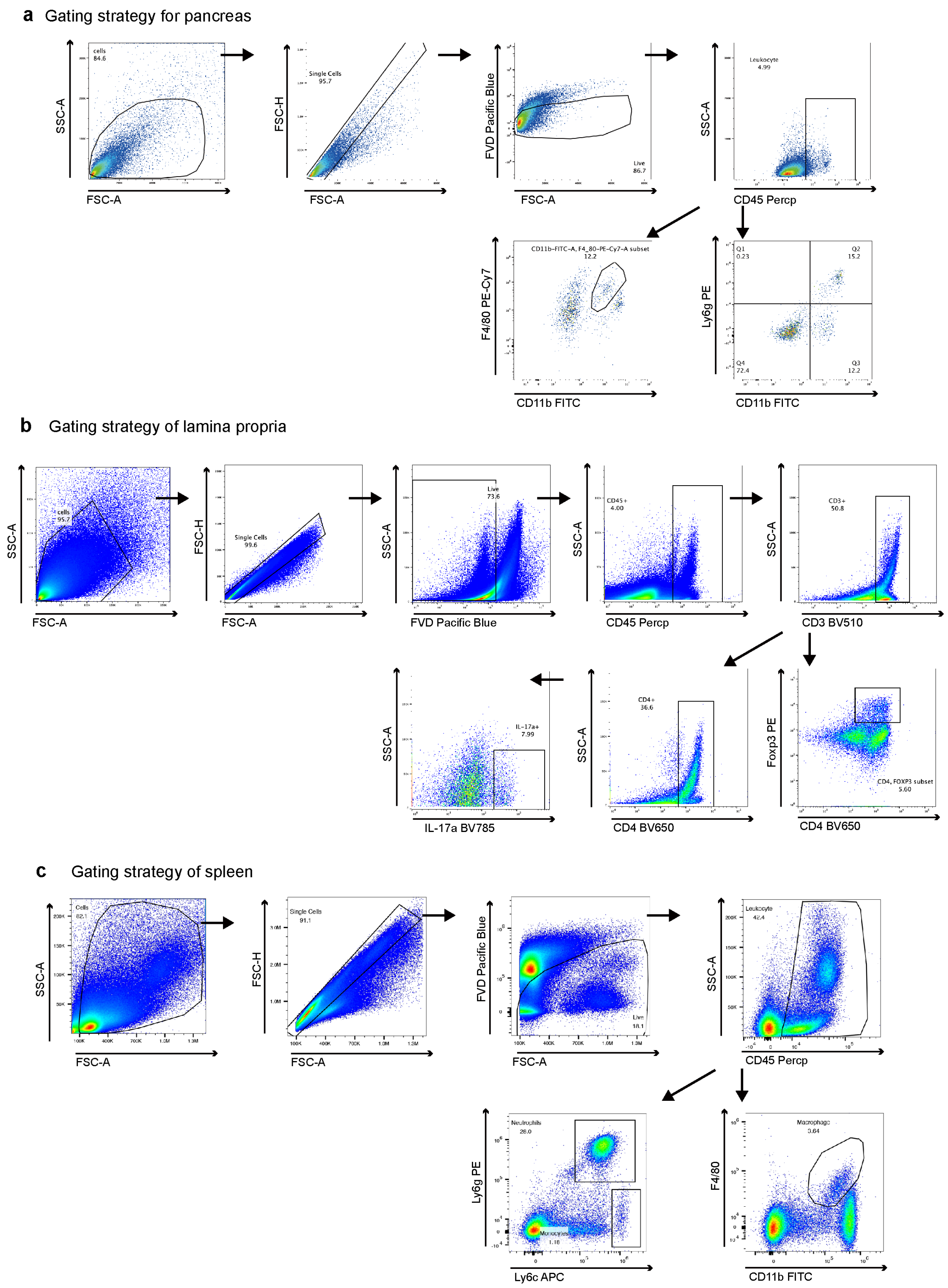

Supplementary Fig.11 Flow cytometry gating strategy.

a. FACS plot showing gating scheme for identification of neutrophils and macrophages in the pancreas. **b.** FACS plot showing gating scheme for identification of Th17 and Treg cells in the lamina propria of colon. **c.** FACS plot showing gating scheme for identification of neutrophils, macrophages and monocytes in the spleen.

Supplementary Table 1.

Table 1. Demographic and clinical characteristics of three groups.

Characteristics	HC(n=16)	HTG(n=8)	HTGP(n=25)	*P* value
Male, n (%)*	9(56.25)	4(50.00)	14(56.25)	1.000
Age(years), mean (SD)#	40.94(±12.80)	41.38(±7.95)	39.48(±10.19)	0.871
Triglyceride(mmol/L),mean (SD)##	1.64(±0.88)	9.14(±2.41)	8.87(±2.86)	<0.001†
BMI (kg/m^2), mean (SD)#	23.87(±2.47)	28.05(±1.34)	27.96 (±2.97)	<0.001†
Drinking, n (%)*	5(31.25)	3(37.50)	9(36.00)	1.000
Smoking, n (%)*	5(31.25)	2(25.00)	8(32.00)	1.000
Hypertension, n (%)*	5(31.25)	2(25.00)	7(28.00)	0.902
Diabetes, n (%)*	6(37.50)	3(37.50)	11(44.00)	0.926

* Fisher's exact test; # Two-tailed ordinary one-way ANOVA; ## Nonparametric rank sum test

† Indicated HC group was significantly different from the other two groups.

Supplementary Table 2.

**Table 2. Demographic and clinical characteristics of FMT donors.**				
Characteristics	HC(n=6)	HTG(n=6)	HTGP(n=6)	*P* value
Male, n (%)*	3(50.00)	3(50.00)	3(50.00)	1.000
Age(years), mean (SD)#	41.83(±7.94)	43.83(±7.47)	42.50(±8.46)	0.902
Triglyceride(mmol/L),mean (SD)##	1.70(±0.81)	9.03(±2.58)	9.59(±2.73)	0.003†
BMI (kg/m^2), mean (SD)##	24.70(±2.78)	28.18(±1.44)	28.82 (±3.10)	0.046†
Drinking, n (%)*	3(50.00)	2(33.33)	3(50.00)	1.000
Smoking, n (%)*	2(33.33)	2(33.33)	1(16.67)	1.000
Hypertension, n (%)*	1(16.67)	2(33.33)	2(33.33)	1.000
Diabetes, n (%)*	4(66.67)	3(50.00)	3(50.00)	1.000

* Fisher's exact test; # Two-tailed ordinary one-way ANOVA; ## Nonparametric rank sum test

† Indicated HC group was significantly different from the other two groups.

Supplementary Table 3. The detailed description and plot visualization of RNA-seq quality control.

sample	raw_reads	clean_reads	clean_bases	error_rate	Q20	Q30	GC_pct
FMT_HTGP1	45916392	44700788	6.71G	0.02	98.15	94.56	50.97
FMT_HTGP2	45222596	43420126	6.51G	0.02	98.27	94.9	51.69
FMT_HTGP3	45502504	44132484	6.62G	0.02	98.19	94.67	51.83
FMT_HC1	46469050	45012728	6.75G	0.02	98.15	94.57	51.82
FMT_HC2	50990750	49785764	7.47G	0.03	97.96	94.19	51.12
FMT_HC3	46102042	44806932	6.72G	0.02	98.23	94.77	51.75

sample	mean	standard_deviation
FMT_HTGP1	287.08306	74.345658
FMT_HTGP2	271.342817	87.35411
FMT_HTGP3	276.578827	78.007395
FMT_HC1	277.836122	81.058038
FMT_HC2	292.111959	77.930104
FMT_HC3	285.72039	82.118185

Supplementary Table 4. The detailed description and plot visualization of ScRNA-seq quality control.

HC group(n=3)	
Demultiplexing	
Raw Reads	1,284,230,825
Valid Reads	1,210,672,145(94.27%)
Q30 of Barcodes	94.78%
Q30 of UMIs	93.25%
**Trimming**	
Reads with Adapters	356,493,393(29.45%)
Reads Too Short	64,809,806(5.35%)
Reads Written	1,145,862,339(94.65%)
Base Pairs Processed	181,600,821,750
Base Pairs Quality-Trimmed	2,851,011,337(1.57%)
Base Pairs Written	149,951,897,819(82.57%)
**Mapping**	
Genome	mus_musculus_ensembl_92
Uniquely Mapped Reads	896,086,721(78.2%)
Multi-Mapped Reads	144,028,812(12.57%)
**FeatureCounts**	
Feature Type	Exon
Reads Assigned To Exonic Regions	758,480,250(84.64%)
Reads Assigned To Intronic Regions	48,486,713(5.41%)
Reads Assigned To Intergenic Regions	67,693,321(7.55%)
Reads Unassigned Ambiguity	21,426,437(2.39%)
**Cells**	
Estimated Number of Cells	11,019
Fraction Reads in Cells	42.00%
Mean Reads per Cell	109,871
Median UMI per Cell	8,685
Total Genes	28,994
Median Genes per Cell	2,590
Saturation	50.69%

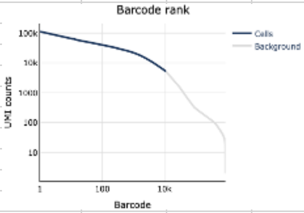

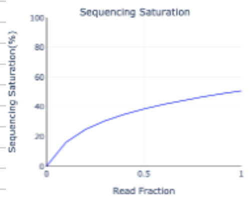

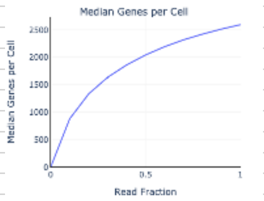

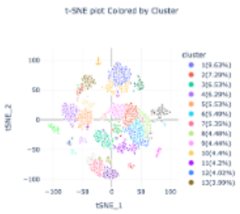

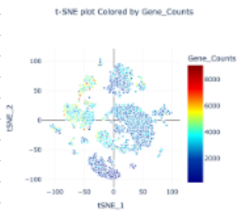

HTGP group(n=3)	
Demultiplexing	
Raw Reads	1,425,883,582
Valid Reads	1,339,825,785(93.96%)
Q30 of Barcodes	94.83%
Q30 of UMIs	93.40%
**Trimming**	
Reads with Adapters	413,946,205(30.9%)
Reads Too Short	85,955,000(6.42%)
Reads Written	1,253,870,785(93.58%)
Base Pairs Processed	200,973,867,750
Base Pairs Quality-Trimmed	3,128,969,658(1.56%)
Base Pairs Written	162,170,136,972(80.69%)
**Mapping**	
Genome	mus_musculus_ensembl_92
Uniquely Mapped Reads	964,835,168(76.95%)
Multi-Mapped Reads	149,831,891(11.95%)
**FeatureCounts**	
Feature Type	Exon
Reads Assigned To Exonic Regions	800,695,576(82.99%)
Reads Assigned To Intronic Regions	66,307,314(6.87%)
Reads Assigned To Intergenic Regions	78,784,044(8.17%)
Reads Unassigned Ambiguity	19,048,234(1.97%)
**Cells**	
Estimated Number of Cells	13,523
Fraction Reads in Cells	42.42%
Mean Reads per Cell	99,077
Median UMI per Cell	9,607
Total Genes	29,998
Median Genes per Cell	2,907
Saturation	44.47%

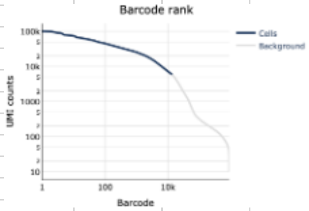

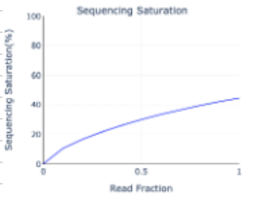

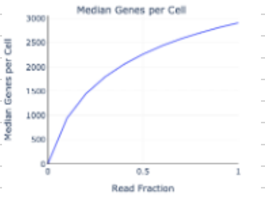

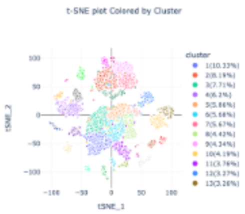

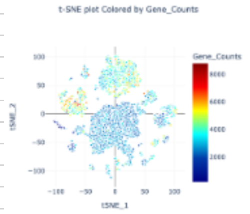

Supplementary Table 5. Primer sequences for Real-time PCR on mice.

Target genes		Sequences
Padi4	Forward	5'- ACGCTGCCTGTGGTCTTTGACT -3'
	Reverse	5'- ACCTCCAGGTTCCCAAAGGCAT -3'
Il17a	Forward	5'- CAGACTACCTCAACCGTTCCAC -3'
	Reverse	5'- TCCAGCTTTCCCTCCGCATTGA -3'
*Nfkb p6*5	Forward	5'- TCCTGTTCGAGTCTCCATGCAG -3'
	Reverse	5'- GGTCTCATAGGTCCTTTTGCGC -3'
Gpihbp1	Forward	5'- CAGCAAACCCTTCTGCATCACG -3'
	Reverse	5'- AGTGTGGACTGGCAACAGGTCT -3'
Gapdh	Forward	5'- CATCACTGCCACCCAGAAGACTG -3'
	Reverse	5'- ATGCCAGTGAGCTTCCCGTTCAG -3'
