# Supplementary material for: Gut microbiota aggravates neutrophil extracellular traps-induced pancreatic injury in hypertriglyceridemic pancreatitis
Source: Nat Commun. 2023 Oct 4;14:6179. doi: 10.1038/s41467-023-41950-y (PMC10550972; doi:10.1038/s41467-023-41950-y)
Supplement: Supplementary file 3 — Reporting Summary [file 41467_2023_41950_MOESM3_ESM.pdf]

nature portfolio

Corresponding author(s): Bei Sun

Last updated by author(s): Sep 10, 2023

Reporting Summary

Nature Portfolio wishes to improve the reproducibility of the work that we publish. This form provides structure for consistency and transparency in reporting. For further information on Nature Portfolio policies, see our Editorial Policies and the Editorial Policy Checklist.

Statistics

For all statistical analyses, confirm that the following items are present in the figure legend, table legend, main text, or Methods section.

n/a	Confirmed	
☐	☒	The exact sample size (*n*) for each experimental group/condition, given as a discrete number and unit of measurement
☐	☒	A statement on whether measurements were taken from distinct samples or whether the same sample was measured repeatedly
☐	☒	The statistical test(s) used AND whether they are one- or two-sided<br>*Only common tests should be described solely by name; describe more complex techniques in the Methods section.*
☐	☒	A description of all covariates tested
☒	☐	A description of any assumptions or corrections, such as tests of normality and adjustment for multiple comparisons
☐	☒	A full description of the statistical parameters including central tendency (e.g. means) or other basic estimates (e.g. regression coefficient) AND variation (e.g. standard deviation) or associated estimates of uncertainty (e.g. confidence intervals)
☐	☒	For null hypothesis testing, the test statistic (e.g. *F*, *t*, *r*) with confidence intervals, effect sizes, degrees of freedom and *P* value noted<br>*Give P values as exact values whenever suitable.*
☒	☐	For Bayesian analysis, information on the choice of priors and Markov chain Monte Carlo settings
☐	☒	For hierarchical and complex designs, identification of the appropriate level for tests and full reporting of outcomes
☐	☒	Estimates of effect sizes (e.g. Cohen's *d*, Pearson's *r*), indicating how they were calculated

Our web collection on statistics for biologists contains articles on many of the points above.

Software and code

Policy information about availability of computer code

Data collection	The 16S rRNA gene sequencing and RNA transcriptomes were collected by Illumina NovoSeq. Metabolomic analysis were collected by UPLC-MS/MS (Vanquish UHPLC system,Thermo Fisher). Targeted amino acid profiling were conducted by GC-MS system (QTRAP 6500). Flow cytometry carried out on BD FACS Celesta (BD Biosciences, San Jose, CA) . Schematic diagram of experimental designs were drawn with Adobe illustrator 2022. Other data were collected with GraphPad Prism software (9.0).
Data analysis	The following software was used for data analysis: GraphPad Prism 9.0, Python (v2.7.9), Scipy (v0.15.1), QIIME2 (202006), R software (V.3.0.1), ImageJ (v1.51), Seurat(4.0), Matplotlib (v1.4.3), Numpy (v1.9.2), FlowJo (10.8).

For manuscripts utilizing custom algorithms or software that are central to the research but not yet described in published literature, software must be made available to editors and reviewers. We strongly encourage code deposition in a community repository (e.g. GitHub). See the Nature Portfolio guidelines for submitting code & software for further information.

Data

Policy information about availability of data

All manuscripts must include a data availability statement. This statement should provide the following information, where applicable:

- Accession codes, unique identifiers, or web links for publicly available datasets
- A description of any restrictions on data availability
- For clinical datasets or third party data, please ensure that the statement adheres to our policy

A data availability statement is included in the manuscript. All 16S rRNA gene sequences were provided and available at National Center for Biotechnology

Information Sequence Read Archive (SRP) database with accession code PRJNA950569 [https://www.ncbi.nlm.nih.gov/bioproject/PRJNA950569]. The metagenomics data, RNA sequencing and single-cell data have been deposited in the Genome Sequence Archive in National Genomics Data Center, China National Center for Bioinformation/Beijing Institute of Genomics, Chinese Academy of Sciences (BioProject: PRJCA016154) that are publicly accessible at [https://ngdc.cncb.ac.cn/bioproject/browse/PRJCA016154]. The raw data are provided as a Source Data file.

Research involving human participants, their data, or biological material

Policy information about studies with human participants or human data. See also policy information about sex, gender (identity/presentation), and sexual orientation and race, ethnicity and racism.

Reporting on sex and gender	Total 49 participants including 25 HTGP patients, 16 healthy volunteers (HC) and 8 high triglycerides (HTG) controls were included in the study. Number of participants for each of the groups analyzed given in Supplementary Table 1. Data was reported disaggregated for sex and gender where this information has been collected and consent has been obtained for reporting and sharing individual-level data, including 27 males and 22 females. Difference between male and female participants were not analyzed due to small group sizes.
Reporting on race, ethnicity, or other socially relevant groupings	The study didn't involve race, ethnicity, or other socially relevant groupings.
Population characteristics	For 16S rRNA sequencing, fecal samples were collected from 25 HTGP patients, 16 healthy volunteers (HC), 8 high triglycerides (HTG) controls and 20 other types of AP patients. Several covariates were considered in the study and are reported in Supplementary Table 1. Beyond BMI and triglycerides level, the clinical characteristics showed no difference between groups.
Recruitment	The inclusion criteria included a diagnosis of AP according to the 2012 revised Atlanta criteria. HTGP was defined as serum triglycerides >1000mg/dL or the serum TG levels between 500 to 1000 mg/dL accompanied by chylous fasting serum after excluding other etiologies of AP. Patients must be presented in the hospital within 24h after disease onset. The major exclusion criteria were chronic pancreatitis, inflammatory bowel disease, cancer, irritable bowel syndrome, gastroenteritis, and use of antibiotics, probiotics or laxatives within two months of enrollment. Finally, 16S rRNA sequencing was performed for fecal samples from 25 HTGP patients, 16 healthy volunteers (HC), 8 high triglycerides (HTG) controls and 20 other types of AP patients.
Ethics oversight	The clinical trial was approved by the Research Ethics Committee of the First Affliated Hospital of Harbin Medical University (IRB-AF/SC-05/05.0).

Note that full information on the approval of the study protocol must also be provided in the manuscript.

Field-specific reporting

Please select the one below that is the best fit for your research. If you are not sure, read the appropriate sections before making your selection.

[x] Life sciences [ ] Behavioural & social sciences [ ] Ecological, evolutionary & environmental sciences

For a reference copy of the document with all sections, see nature.com/documents/nr-reporting-summary-flat.pdf

Life sciences study design

All studies must disclose on these points even when the disclosure is negative.

Sample size	Sample sized were determined based on pilot studies, or according to prior experience with similar studies. Animal's number calculation was by "resource equation" method. Based on this method, a value "E" is measured. The value of E should lie between 10 and 20. If E is less than 10 then adding more animals will increase the chance of getting more significant results, but if it is more than 20 then adding more animals will not increase the chance to get significant results. It is considerable to be applicable to all animal experiments. Any sample size, which keeps E between 10 and 20 should be considered as an adequate. E can be measured by following formula: E=Total number of animals-Total number of groups.
Data exclusions	No data were excluded.
Replication	All experimental results were pooled from three independent experiments. All replication were successful.
Randomization	Human participants were randomly selected under the criteria in the Methods. Six- to eight-week-old mice were randomly divided into indicated experimental groups, with at least 5 mice per group, and the mice did not show differences before treatment.
Blinding	For HE and IHC staining and analysis, pathologists were blinded to group allocation. The investigators were not blinded during group allocation and the experiment.

Reporting for specific materials, systems and methods

We require information from authors about some types of materials, experimental systems and methods used in many studies. Here, indicate whether each material, system or method listed is relevant to your study. If you are not sure if a list item applies to your research, read the appropriate section before selecting a response.

Materials & experimental systems

n/a	Involved in the study
☐	☒ Antibodies
☒	☐ Eukaryotic cell lines
☒	☐ Palaeontology and archaeology
☐	☒ Animals and other organisms
☒	☐ Clinical data
☒	☐ Dual use research of concern
☒	☐ Plants

Methods

n/a	Involved in the study
☒	☐ ChIP-seq
☐	☒ Flow cytometry
☒	☐ MRI-based neuroimaging

Antibodies

Antibodies used

The following primary antibodies were used for IHC. They are listed as antigen first, following by supplier, catalog number and clone/lot number as applicable.
1. Anti-Myeloperoxidase antibody (abcam, clone: EPR20257, ab208670, IHC 1:1000)
2. Ly-6G (E6Z1T) Rabbit mAb (Cell Signaling Technology, 87048s, IHC 1:250)
3. Anti-Histone H3 (citrulline R2 + R8 + R17) antibody (abcam, ab5103, IHC 1:100)

The following primary antibodies were used for IF. They are listed as antigen first, following by supplier, catalog number and clone/lot number as applicable.
1. Human/Mouse Myeloperoxidase/MPO Antibody (R&D, AF3667, IF 1:500)
2. Purified anti-mouse Ly-6G Antibody (BioLegend, clone:1A8, 127602, IF 1:800)
3. CD45 (Intracellular Domain) (D9M8I) XP® Rabbit mAb (Cell Signaling Technology, 13917, IF 1:200)

The following primary antibodies were used for flow cytometry. They are listed as antigen first, following by supplier, catalog number and clone/lot number as applicable. All surface antibodies were used at 1:100 dilution; all intracellular antibodies were used at 1:50 dilution.
1. FVD (Thermo Fisher Scientific, 34955,)
2. PerCP/Cyanine5.5 anti-mouse CD45 Antibody (BioLegend, 103132)
3. Alexa Fluor®488 anti-mouse/human CD11b Antibody (BioLegend, 101217)
4. Brillant Violet 785TM anti-mouse F4/80 Antibody (BioLegend, 123141)
5. PE anti-mouse Ly-6G Antibody (BioLegend, 127607)
6. APC anti-mouse Ly-6c Antibody (BioLegend, 128015)
7. Brillant Violet 510TM anti-mouse CD3 Antibody (BioLegend, 100234)
8. Brillant Violet 650TM anti-mouse CD4 Antibody (BioLegend, 100555)
9. FOXP3 Monoclonal Antibody, PE (Thermo Fisher Scientific 12-5773-82)
10. ROR gamma (t) Monoclonal Antibody, APC (Thermo Fisher Scientific 17-6988-82)
11. Brillant Violet 785TM anti-mouse IL-17a Antibody (BioLegend, 506928)
12. Fc block (Invitrogen 14-0161-81)

The following primary antibodies were used for Western Blot. They are listed as antigen first, following by supplier, catalog number and clone/lot number as applicable.
1. anti-NF-κB p65 (Cell Signaling Technology, #8242, WB1:1000)
2. anti-phospho-NF-κB p65 (Cell Signaling Technology, #3033, WB1:1000)

The following primary antibodies were used for in vivo therapy.
1. InVivoMAb anti-mouse Ly6G (BioXCell, #BE0075-1)
2. Rat IgG2a isotype (BioXCell, #BE0089)

Validation

Commercial available antibodies were selected based on their specificity and suggested appliation as described on the manufacturer's website and data sheets. All antibodies were validated in the literature (listing the PMID of each antibody validated literature).
1. Anti-Myeloperoxidase antibody https://www.abcam.cn/products/primary-antibodies/myeloperoxidase-antibody-epr20257-ab208670.html Validated for IHC (PMID:36425537, PMID:36555773, PMID:36611891)
2. Ly-6G (E6Z1T) Rabbit mAb https://www.cellsignal.cn/products/primary-antibodies/ly-6g-e6z1t-rabbit-mab/87048?site-search-type=Products&N=4294956287&Ntt=87048&fromPage=plp&_requestid=703586 Validated for IHC (PMID:15467490, PMID:8360469, PMID10861067)
3. Anti-Histone H3 (citrulline R2 + R8 + R17) antibody https://www.abcam.cn/products/primary-antibodies/histone-h3-citrulline-r2--r8--r17-antibody-ab5103.html Validated for IHC (PMID: 36453281, PMID:36552941, PMID:36559875)
4. Human/Mouse Myeloperoxidase/MPO Antibody https://www.rndsystems.com/cn/products/human-mouse-myeloperoxidase-mpo-antibody_af3667 Validated for immunofluorescent (PMID: 36827917, PMID:36495695, PMID:36430952)
5. Purified anti-mouse Ly-6G Antibody https://www.biolegend.com/en-us/products/purified-anti-mouse-ly-6g-antibody-4767

Validated for immunofluorescent (PMID: 17884993, PMID:32086318, PMID:211145693)
6. CD45 (Intracellular Domain) (D9M8I) XP® Rabbit mAb https://www.cellsignal.cn/products/primary-antibodies/cd45-intracellular-domain-d9m8i-xp-rabbit-mab/13917?site-search-type=Products&N=4294956287&Ntt=13917&fromPage=plp&_requestid=672877 Validated for immunofluorescent (PMID: 36442992, PMID:37377902, PMID:36700195)
7. FVD https://www.thermofisher.cn/order/catalog/product/L34955?SID=srch-srp-L34955 Validated for flow cytometry (PMID: 18836993, PMID:18453600, PMID:19235202)
8. PerCP/Cyanine5.5 anti-mouse CD45 Antibody https://www.biolegend.com/en-us/products/percp-cyanine5-5-anti-mouse-cd45-antibody-4264 Validated for flow cytometry (PMID: 33440159, PMID:34363749, PMID:35361799)
9. Alexa Fluor®488 anti-mouse/human CD11b Antibody https://www.biolegend.com/en-us/products/alexa-fluor-488-anti-mouse-human-cd11b-antibody-2700 Validated for flow cytometry (PMID: 30926232, PMID:35641483, PMID:33826903)
10. Brillant Violet 785TM anti-mouse F4/80 Antibody https://www.biolegend.com/en-us/products/brilliant-violet-785-anti-mouse-f4-80-antibody-9919 Validated for flow cytometry (PMID: 31661465, PMID:30076102, PMID:32438752)
11. PE anti-mouse Ly-6G Antibody https://www.biolegend.com/en-us/products/pe-anti-mouse-ly-6g-antibody-4777 Validated for flow cytometry (PMID: 32086318, PMID:32034922, PMID:31089128)
12. APC anti-mouse Ly-6c Antibody https://www.biolegend.com/en-us/products/apc-anti-mouse-ly-6c-antibody-6047 Validated for flow cytometry (PMID:32034922, PMID:33333014, PMID:31375667)
13. Brillant Violet 510TM anti-mouse CD3 Antibody https://www.biolegend.com/en-us/products/brilliant-violet-510-anti-mouse-cd3-antibody-7990 Validated for flow cytometry (PMID:35864538, PMID:33979608, PMID:33789089)
14. Brillant Violet 650TM anti-mouse CD4 Antibody https://www.biolegend.com/en-us/products/brilliant-violet-650-anti-mouse-cd4-antibody-7634 Validated for flow cytometry (PMID:32160524, PMID:36300116, PMID:31027998)
15. FOXP3 Monoclonal Antibody, PE https://www.thermofisher.cn/cn/zh/antibody/product/FOXP3-Antibody-clone-FJK-16s-Monoclonal/12-5773-82 Validated for flow cytometry (PMID:36763100, PMID:36657993, PMID:36849508)
16. ROR gamma (t) Monoclonal Antibody, APC https://www.thermofisher.cn/cn/zh/antibody/product/ROR-gamma-t-Antibody-clone-AFKJS-9-Monoclonal/17-6988-82 Validated for flow cytometry (PMID:34910890, PMID:34312491, PMID:30197303)
17. Brillant Violet 785TM anti-mouse IL-17a Antibody https://www.biolegend.com/en-us/products/brilliant-violet-785-anti-mouse-il-17a-antibody-7988 Validated for flow cytometry (PMID:36389710, PMID:33986290)
18. Fc block https://www.thermofisher.cn/cn/zh/antibody/product/CD16-CD32-Antibody-clone-93-Monoclonal/14-0161-81 Validated for flow cytometry (PMID:36642403, PMID:36917181, PMID:36755018)
19. anti-NF-κB p65 https://www.cellsignal.cn/products/primary-antibodies/nf-kb-p65-d14e12-xp-rabbit-mab/8242?site-search-type=Products&N=4294956287&Ntt=%238242&fromPage=plp&_requestid=707904 Validated for flow cytometry (PMID:37477163, PMID:37464262, PMID:37460545)
20. anti-phospho-NF-κB p65 https://www.cellsignal.cn/products/primary-antibodies/phospho-nf-kb-p65-ser536-93h1-rabbit-mab/3033?site-search-type=Products&N=4294956287&Ntt=%233033&fromPage=plp&_requestid=708329 Validated for flow cytometry (PMID:37468960, PMID:37464262, PMID:37427330)
21. InVivoMAb anti-mouse Ly6G https://bioxcell.com/invivomab-anti-mouse-ly6g-be0075-1 Validated for invivo therapy (PMID:30357853, PMID:27775706, PMID:26070485)
22. Rat IgG2a isotype https://bioxcell.com/invivomab-rat-igg2a-isotype-control-anti-trinitrophenol-be0089 Validated for invivo therapy (PMID:30097293, PMID:26208901, PMID:25142145)

Animals and other research organisms

Policy information about studies involving animals; ARRIVE guidelines recommended for reporting animal research, and Sex and Gender in Research

Laboratory animals	C57BL/6 mice (male, 6-8 weeks old) were purchased from Cyagen Biosciences Inc. (Suzhou, China). GPIHBP1-/- mice and PAD4-/- mice, were purchased from Cyagen Biosciences Inc. Age- and sex-matched wild-type C57BL/6N mice were used as controls. All animals were housed in specific pathogen-free environment with controlled conditions, including a constant temperature (22±2 °C), 12-h light/dark cycle and 50 ± 5% humidity. The animals had free access to water and standard chow (Xie Tong Biotechnology, SFS9112). The sterile drinking water was replaced twice a week. The above animals were commercially available and acclimated for at one week before experiment started. All experimental procedures involving animals were carried out according to protocols approved by the Institutional Animal Ethics Committee of The First Affiliated Hospital of Harbin Medical University.
Wild animals	The study did not include wild animals.
Reporting on sex	All the wild type mice used in fecal microbiota transplantation study were from same gender (male). The gene knockout mice, including GPIHBP1-/- mice and PAD4-/- mice, were employed by sex to allow an approximately equal number of females and males. Age- and sex-matched wild-type mice were used as controls.
Field-collected samples	The study did not include samples collected from fields.
Ethics oversight	All experimental procedures involving animals were carried out according to protocols approved by the Institutional Animal Ethics Committee of The First Affiliated Hospital of Harbin Medical University (IACUC number:2020010).

Note that full information on the approval of the study protocol must also be provided in the manuscript.

Flow Cytometry

Plots

Confirm that:

- [x] The axis labels state the marker and fluorochrome used (e.g. CD4-FITC).
- [x] The axis scales are clearly visible. Include numbers along axes only for bottom left plot of group (a 'group' is an analysis of identical markers).
- [x] All plots are contour plots with outliers or pseudocolor plots.
- [x] A numerical value for number of cells or percentage (with statistics) is provided.

Methodology

Sample preparation

At the end of HTGP induction, the mice were euthanized, and the tissues (pancreas, colon and spleen) were collected.

1.The pancreatic tissue was resuspended in buffer A containing 2 mg/mL collagenase type IV (Sigma-Aldrich) and incubated in a shaker at 37°C for 15 minutes. The suspension was then vortexed at low speed for 20 seconds and centrifuged, and the cell pellet was resuspended in red blood cell lysing buffer for 5 minutes.
Cells were incubated with Fc block and stained in different combinations of fluorochrome-conjugated antibodies. For surface markers staining, cells were stained with FVD (Thermo Fisher Scientific, Cat.L34955), CD45 (BioLegend, Cat.103132), CD11b (BioLegend, Cat.101217), F4/80 (BioLegend, Cat.123141), Ly6g (BioLegend, Cat.127607), Ly6c (BioLegend, Cat.128015) antibodies.

2.Splenocytes were separated through a 70 μm cell strainer.
After washing and lysis of erythrocytes, cells were incubated with Fc block and stained in different combinations of fluorochrome-conjugated antibodies. For surface markers staining, cells were stained with FVD (Thermo Fisher Scientific, Cat.L34955), CD45 (BioLegend, Cat.103132), CD11b (BioLegend, Cat.101217), F4/80 (BioLegend, Cat.123141), Ly6g (BioLegend, Cat.127607), Ly6c (BioLegend, Cat.128015) antibodies. antibodies.

3.Intestinal lamina propria cells were isolated using 2 mg/mL collagenase D (Roche, Basel, Switzerland), 1 mg/mL dispase (Invitrogen, Carlsbad, CA), and 15 mg/mL DNase I for 60 minutes in a 37°C shaking water bath for flow cytometry. Cells were incubated with Fc block and stained in different combinations of fluorochrome-conjugated antibodies.
Cells were stained with FVD (Thermo Fisher Scientific, Cat.L34955), CD45 (BioLegend, Cat.103132), CD3 (BioLegend, Cat.100234), CD4 (BioLegend, Cat.10555), FOXP3 (Thermo Fisher Scientific, Cat.12-5773-82), ROR gamma (t) (Thermo Fisher Scientific, Cat.17-6988-82), anti-mouse IL-17A (BioLegend, Cat.506928) antibodies. After fixation and permeabilization cells were stained for the transcription factors (BioLegend, Cat.424401). For intracellular staining, cells were fixed and permeabilized with the eBioscience Foxp3/Transcription Factor Staining Buffer Set.

Instrument

Sample acquisition was carried out on BD FACS Celesta (BD Biosciences, San Jose, CA)

Software

Data analysis was performed by FlowJo software 10.8.

Cell population abundance

Each cell frequency in pancreas was determined by Flow cytometry assay. The total number of cells were counted using hemocytometer for cells from each group. The abundance of each population was determined by multiplying cell frequency of total cell number for each sample.

Gating strategy

Gating strategies for flow cytometry analysis have been deposited in Supplementary Fig.11.

- [x] Tick this box to confirm that a figure exemplifying the gating strategy is provided in the Supplementary Information.
